# In Situ Genomics and Transcriptomics of SAR202 Subclusters Revealed Subtle Distinct Activities in Deep-Sea Water

**DOI:** 10.3390/microorganisms10081629

**Published:** 2022-08-12

**Authors:** Zhanfei Wei, Qingmei Li, Rui Lu, Pengfei Zheng, Yong Wang

**Affiliations:** 1 Institute of Deep-Sea Science and Engineering, Chinese Academy of Sciences, Sanya 572000, China; 2 Yellow Sea Fisheries Research Institute, Chinese Academy of Fishery Sciences, Qingdao 266071, China; 3 Shenzhen International Graduate School, Tsinghua University, Shenzhen 518055, China

**Keywords:** microbial community, SAR202, DOM, omics data, MISNAC

## Abstract

Deep-sea water columns are enriched with SAR202 that may conduct detrital matter degradation. There are several subclusters in SAR202, but their subtle differences in geochemical cycles are largely unknown, particularly for their in situ activities in the marine deep zone. Deep-sea DNA/RNA samples obtained from 12 continuous time periods over two days by in situ nucleic acid collection apparatus were used to re-evaluate the ecological functions of each SAR202 subcluster at a depth of ~1000 m in the South China Sea (SCS). Phylogenomics of 32 new SAR202 genomes from the SCS and western Pacific revealed their distribution in five subclusters. Metatranscriptomics analysis showed that the subclusters II and III were the dominant SAR202 groups with higher transcriptional activities in the SCS deep-sea zone than other subclusters. The analyses of functional gene expression further indicated that SAR202 subclusters II and III might be involved in different metabolic pathways in the deep-sea environment. The SAR202 subcluster III might take part in the degradation of deep-sea aromatic compounds. Time-course metagenomics and metatranscriptomics data did not show metabolic correlation of subclusters II and III over two days, suggesting diversified ecological functions of SAR202 subclusters under different organic inputs from the overlying water column. Collectively, our results indicate that the SAR202 subclusters play different roles in organic degradation and have probably undergone subtle and gradual adaptive evolution in the dynamic environment of the deep ocean.

## 1. Introduction

As the largest habitat on earth, the ocean is full of mysteries and unknowns. Various microbial assemblies that play important roles in biogeochemical cycles have been found in different water layers of the ocean [1], which can be divided into different zones with significantly different microbial communities [2]. The aphotic zone below a depth of 200 m is characterized by low temperature and oligotrophy, where microbial inhabitants depend on the organic matter transported from the surface by a ‘biological pump’ [3]. Deep-sea microorganisms can even survive in the dark and in an extremely high hydrostatic pressure environment up to ~11,000 m below the sea surface [4,5]. With various environmental factors and geographical patterns, the deep-water layers are remarkably dynamic, which promotes the diversification of ecosystems and formation of ecotypes [6,7]. Although seawater samples have been collected at different depths and locations to reveal the diversity and physiological and genetic characteristics of marine microorganisms, the traditional sampling methods cannot meet the needs of capturing the subtle changes in the deep-sea ecosystem and ecotypes [8,9,10,11,12]. Moreover, most of the microorganisms in deep-sea water are not cultured and the sampling method may result in alterations to the microbial community and in situ activities. Previous studies found significant differences in functional gene expressional patterns between the traditional sampling device Niskin bottles and in situ filtration apparatus in the deep sea [9,12]. Studies of the hadal water samples found that the in situ samples obtained by an in situ microbial filtration and fixation (ISMIFF) apparatus could detect more microbes and avoid microbial disturbance in the upper layers [13]. Our previous in situ metatranscriptomic data indicated that the RNA fragments could be better preserved by MISNAC, which could avoid degradation during sampling [11]. Therefore, the differences in the metabolic activities of deep-sea ecotypes are mostly undetectable, and most of the ‘dark matter’ microorganisms in the deep ocean cannot be evaluated in terms of their real contribution to the geochemical cycle without in situ experiments and omics data.

SAR202, belonging to the Chloroflexota phylum, was identified by 16S rRNA gene clone sequencing in Sargassum in 1996 [14], and is widely and abundantly distributed in the global marine environment [15]. The SAR202 genomes vary in genome size and have been divided into multiple subclusters, which indicates the plasticity of their genomes [16]. In addition, SAR202 cells could participate in the degradation of refractory, dissolved organic matter (DOM) and be involved in sulfur metabolism in the ocean [15,17,18,19]. Analysis of the vertical distribution, genomic characteristics and metabolic potentials of SAR202 in the Mariana Trench suggested that they play important roles in the carbon and sulfur cycles, and are the most active microbial group in the hadal zone where DOM is enriched [5,15].

Previous studies have assessed the ecological roles of SAR202 subclusters at the metagenomic and genomic levels [15,17,18,19]. However, the limitation of deep-sea sampling technology and lack of deep-sea in situ nucleic acid samples introduced difficulties in revealing the in situ activities of each SAR202 subcluster in the deep ocean, which might have hindered the assessment of the ecological contribution and limiting factors of SAR202 subclusters in the deep-sea environment. In this study, we conducted time-course in situ deep-sea nucleotide collections in 12 temporal scales using multiple in situ nucleic acid collections (MISNAC) apparatus in the South China Sea (SCS). The relative abundance, transcriptional activities and metabolic potentials of each SAR202 subcluster were analyzed based on the in situ metagenomics and metatranscriptomics data. We found that the prevalence of SAR202 subclusters varied at different time periods, and they were actively involved in different metabolic pathways. This is evidence for the subtle and gradual adaptive evolution of SAR202 subclusters to participate in different processes of material cycles in the deep-sea environment.

## 2. Material and Methods

### 2.1. In Situ Nucleic Acid Extraction

**Figure 1:**
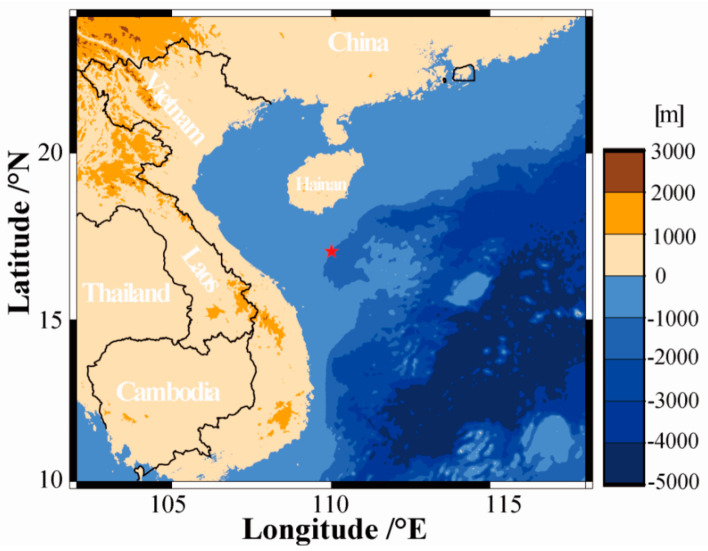
Deployment locations of ‘Phoenix’ lander in the SCS. The red star denotes the site where in situ DNA/RNA co-collection was conducted by MISNAC apparatus in 12 time periods of two days in November 2019 at 1022 m depth (Table S1 for more details).

A total of 12 day-to-night continuous DNA/RNA co-collection samples were obtained by the MISNAC apparatus carried by ‘Phoenix’ lander during the cruise in November 2019 at 1022 m depth in the SCS (Figure 1 and Table S1). The physical parameters of MISNAC and the preparation of MISNAC deployment were introduced in Wei et al. [11]. In short, the deep-sea water was prefiltered by a 1-mm pore size filter before entering the chamber, which contained the 0.22 μm polycarbonate membranes (Millipore, Bedford, MA, USA) with 142 mm diameter, and then the DNA/RNA co-collection adsorption columns (Tiangen, Beijing, China) were installed in the nucleic acid collection subsystem of MISNAC. The MISNAC apparatus completed the in situ nucleic acid sample collection of 12 working units in two days (4-h separation between two deployments); each working unit lasted four hours to accomplish 80 L seawater filtration, microbial enrichment, cell lysis and DNA/RNA co-collection. When onboard the MISNAC apparatus, the nucleic acid adsorption columns were processed in the shipboard laboratory following the steps: (i) the nucleic acid adsorption columns were centrifuged at 6000 rpm for one minute; (ii) the GD and PW buffers (TIANamp Genomic DNA Kit, Tiangen, Beijing, China) were used to remove residual salt and ethanol; (iii) the nucleic acids attached in the columns were washed with DNase/RNase-Free water (Tiangen, Beijing, China) by centrifuge at 6000 rpm. The 12 deep-sea in situ DNA/RNA samples were quantified by Qubit 2.0 Fluorometer (Life, Carlsbad, CA, USA) onboard, and were then immediately transferred to −80 °C for subsequent experiments. In addition, a conductivity, temperature and depth (CTD) (Sea-Bird, Bellevue, WA, USA) sensor was equipped on the ‘Phoenix’ lander to monitor deep-sea environmental factors including dissolved oxygen during the sampling work.

### 2.2. Omics Sequencing

A total of 12 MISNAC nucleic acid samples were separately incubated with 0.1 ng/μL RNaseA (Takara, Dalian, China) at 37 °C for 10 min to remove RNA fragments. Due to the low concentration of DNA, VAHTS DNA clean beads (Vazyme, Nanjing, China) were used for DNA concentration. About 100 ng DNA was fragmented to 350 bp by ultrasound with Covaris M220 (Covaris, Woburn, MA, USA), and then the high-throughput metagenome libraries were constructed by using VAHTS universal DNA library preparation kit for Illumina V3 (Vazyme, Nanjing, China) according to the manufacturer’s protocol. Moreover, DNA fragments were removed from 12 MISNAC nucleic acid samples using Turbo DNA-free^TM^ kit (Ambion, Carlsbad, CA, USA) according to the instruction. The universal primers of the V3-V4 variable region of 16S rRNA genes (341F: 5′-CCTAYGGGRBGCASCAG-3 and 802R: 5′-TACNVGGGTATCTAATCC-3′) were used for PCR amplification to ensure the removal of DNA in nucleic acid samples [20,21]. Double-stranded cDNA was synthesized from 5 ng RNA using the Ovation RNA-Seq System V2 Kit (Qiagen, Hildon, Germany) according to the instruction. VAHTS universal DNA library preparation kit for Illumina V3 (Vazyme, Nanjing, China) was used to construct cDNA libraries. The DNA and cDNA libraries were sequenced on an Illumina NovaSeq6000 platform (Illumina, San Diego, CA, USA) with paired-end 2 × 150 bp.

### 2.3. Data Analysis

The raw data (Table S1) from metagenomic sequencing were assessed by Fastqc (v0.11.9) (Simon Andrews, Cambridge, UK) with default settings [22]. The low quality reads (QC < 20 over 20% of the reads), reads less than 50 bp in length and adaptors were removed using Fastp (v0.21.0) (Shifu Chen, Shenzhen, China) (‘-f 5 -F 5 -q 20 -u 20 -g -W 5 -3 -l 50’) [23]. The FastUniq (Haibin Xu, Nanjing, China) softwarewas used to remove the duplicates in paired short reads with the default parameters [24]. The 12 MISNAC clean data from metagenomic sequencing were assembled using MEGAHIT (Dinghua Li, Hongkong, China) software (‘-min-contig-len 300 -m 0.9 -k-min 21 -k-max 141 -k-step 10’) [25], respectively, according to different sampling methods. The MetaWRAP (v1.2.1) (Gherman Uritskiy, Baltimore, MD, USA) software was used to select the MAGs with completeness greater than 50% and contamination less than 10% (metawrap bin_refinement ‘-c 50 -x 10’) [26]. De-redundancy of the MAGs obtained by MISNAC were processed using the dRep (Matt Olm, Berkeley, CA, USA) software (‘-comp 50 -con 10 -p 28 -l 1000’) [27]. The GTDB-TK (v1.0.2) (Pierre-Alain Chaumeil, St. Lucia, Australia) software was used to classify the obtained non-redundant MAGs [28]. Finally, the MAGs belonging to SAR202 were selected for further analyses.

The Fastp (v0.21.0) software was used to remove the low quality and short reads (QC < 20 over 20% of the reads, or length < 50 bp), adaptors and polyX in metatranscriptomic sequencing raw data with the settings ‘-f 10 -F 10 -q 20 -u 20 -g -W 5 -3 -l 50 -x’ [23]. The reads containing one single base more than 70% of the reads were removed using an in-house script. The redundant data were processed using FastUniq software to delete duplicates with the default parameters [24]. The SortMeRNA (Evguenia Kopylova, LIFL, France) software was used to remove the rRNA reads from 12 MISNAC pre-clean data with e-value less than 1 × 10^−5^ [29].

The 16S rRNA fragments > 100 bp were extracted from metagenomic single clean data and metatranscriptomic single pre-clean data using rRNA_HMM [30]. The 16S rRNA fragments that mapped to V3-V4 regions were selected using hmmsearch for the structural analysis of microbial communities using QIIME1 workflow [31]. The reads that shared similarity level at 97% were clustered to operational taxonomic units (OTUs) using UCLUST [32]. The longest read of each OTU was used as a representative read for further taxonomic classification analysis with the SILVA 132 database by PyNAST and Ribosomal Database Project (RDP v2.2) classifier [33,34]. All the OTUs sorted to chloroplasts, mitochondria and eukaryotes were excluded.

### 2.4. Annotation and Phylogenomics of SAR202 MAGs

Seven SAR202 MAGs in this study were combined with 25 SAR202 MAGs from the western Pacific. The genome completeness and potential contaminations of 32 SAR202 MAGs were examined using CheckM (Donovan H Parks, St. Lucia, Australia) with default settings [35]. The open reading frames (ORFs) were predicted using Prodigal (v2.6.2) (Doug Hyatt, Oak Ridge, TN, USA) (with the parameter ‘-p meta’) in the metagenome mode [36]. The predicted ORFs were annotated by eggNOG (v2.0.1-4) (Jaime Huerta-Cepas, Heidelberg, Germany) with parameter ‘-d bact’ [37]. In addition, prediction of 16S rRNA genes in the MAGs was performed using rRNA_HMM [30].

For phylogenomics analysis, 158 Chloroflexota genomes and two Deinococcota (Table S2) collected from NCBI and IMG databases were pooled with the 32 SAR202 MAGs. A total of 43 concatenated alignment of protein sequences in these genomes were produced by CheckM with default settings [35] and trimmed using trimAl (v1.4) (Salvador Capella-Gutiérrez, Barcelona, Spain) with the parameters ‘-automated1 -fasta’ to remove the poorly aligned regions [38]. A maximum likelihood phylogenomic tree was constructed using IQ-TREE (v1.6.12) (Lam-Tung Nguyen, Vienna, Austria) under ‘MFP’ to select the optimal model with 1000 replicates (‘-m MFP -bb 1000 -alrt 1000’) [39]. Average nucleotide identity (ANI) comparison between the 130 SAR202 genomes was calculated using FastANI (v1.33) (Chirag Jain, Atlanta, GA, USA) [40].

To assess the taxonomic ranks of SAR202 subclusters, the relative evolutionary divergence (RED) approach was used to evaluate taxa at the same taxonomic rank diverged at similar times. Five genomes from each phylum, except for Chloroflexota, in bacteria kingdom were randomly selected using an in-house script from GTDB database and combined with 158 Chloroflexota genomes. The genomes with ≥70% of 120 single-copy conserved proteins (Table S3) were further used to calculate the RED with PhyloRank (Aaron Mussig, St. Lucia, Australia) (v0.0.27; available online: https://github.com/dparks1134/PhyloRank (accessed on 10 August 2021)). A total of 120 single-copy conserved proteins were aligned to Pfam and TIGRfam hidden Markov models (HMMs) using HMMER (v.3.1b2) (L Steven Johnson, St. Louis, MO, USA) with default parameters, and concatenated to construct trees inferred with FastTree (v2.1.7) (Morgan N. Price, Berkeley, CA, USA) under the WAG + GAMMA model [41].

### 2.5. Relative Abundance of the SAR202 Subclusters in Metagenomics and Metatranscriptomics

To explore the distribution characteristics of the SAR202 subclusters at ~1000 m depth in the SCS, 16S rRNA fragments of more than 1000 bp were selected from the genomes of SAR202 subclusters I–VII, and compared with the 16S rRNA fragments from metagenomic single clean data and metatranscriptomic single pre-clean data by BLASTN. The 16S rRNA fragments with length of >100 bp and similarity of >97% were used to calculate the relative abundance of each SAR202 subcluster in metagenomes and metatranscriptomes for the MISNAC samples.

### 2.6. Transcriptional Activities and Gene Expression Patterns of SAR202 Subclusters

The ORFs from 32 SAR202 MAGs were mapped to the 12 MISNAC metatranscriptomic clean data using bowtie2 with setting ‘-very-sensitive-local’ [42]. The number of reads matched to each SAR202 ORF was counted by using the Coverm (v0.3.0) (Ben J Woodcroft, St. Lucia, Australia) software (‘contig -m count -min-read-aligned-percent 0.95 -min-read-percentage-identity 0.95’). Finally, the in situ transcriptional activities of each SAR202 genome and of their functional genes were estimated by counting the reads recruited per kilobase of genome per gigabase of metagenome (RPKG) and transcripts per million reads (TPM), respectively. Averages of TPM values (>1 in three or more transcriptomes) for different time periods were calculated for different SAR202 subclusters or functional genes.

## 3. Results

### 3.1. In Situ Samples’ Collection and Environmental Factors’ Measurement

Our samples were obtained by the ‘Phoenix’ lander carrying MISNAC apparatus at a depth of 1022 m in the SCS (Figure 1). A total of 12 MISNAC nucleic acid samples were obtained in consecutive 4-h periods (Table S1). The CTD sensors equipped on the lander were used to monitor the temperature, dissolved oxygen and salinity in the deep sea (Figure S1). The water temperature and dissolved oxygen decreased rapidly from the sea surface to the bottom, and there was no obvious fluctuation in the water temperature at a depth of ~1000 m with an average value of 4.35 ± 0.04 °C. During the first dive, the dissolved oxygen increased significantly 10 h after deployment, and then gradually decreased to 2.07 ± 0.7 mL/L, probably due to the instability of the sensor or oxygen flux. The salinity did not fluctuate notably with the increase in seawater depth and across different time periods, with an average value of 34.28 ± 0.01 PSU.

### 3.2. Microbial Community in the Metagenomic and Metatranscriptomic Data

Based on the 16S rRNA fragments (miTags) from 12 MISNAC metagenomic data, the microbial community structures in the SCS were analyzed (Figures S2A and S3A). Proteobacteria (67.4% ± 5.9%), Actinobacteria (5.3% ± 1.5%), Bacteroidetes (4.2% ± 2.0%), Firmicutes (4.0% ± 2.8%), Marinimicrobia (3.4% ± 1.2%), Planctomycetes (3.1% ± 1.0%), Chloroflexota (1.4% ± 0.6%) and Acidobacteria (1.4% ± 1.3%) were the main bacterial phyla, while Thaumarchaeota (5.4% ± 2.0%) and Euryarchaeota (2.4% ± 0.5%) were the main archaeal phyla. SAR202 accounted for 1.1 ± 0.5% of the total microbial communities in the MISNAC samples, which was one of the dominant bacteria at the deep-sea site within the two days. In this study, we also analyzed the community compositions in the metatranscriptomic data (Figures S2B and S3B). Proteobacteria (61.6% ± 11.4%) Marinimicrobia (9.2% ± 3.2%), Planctomycetes (7.4% ± 1.6%), Chloroflexota (5.0% ± 1.8%) and Bacteroidetes (1.7% ± 1.0%) were among the most active bacterial phyla. Moreover, Thaumarchaeota (4% ± 5.9%) and Euryarchaeota (67.4% ± 5.9%) were also the major active archaeal phyla. SAR202 accounted for 5.5 ± 0.9% of the active microorganisms in the MISNAC samples, which was significantly higher than their percentages in the metagenomics data (*U*-test; *p* < 0.01).

### 3.3. SAR202 Genome Reconstruction and Affinity Relationships

**Figure 2:**
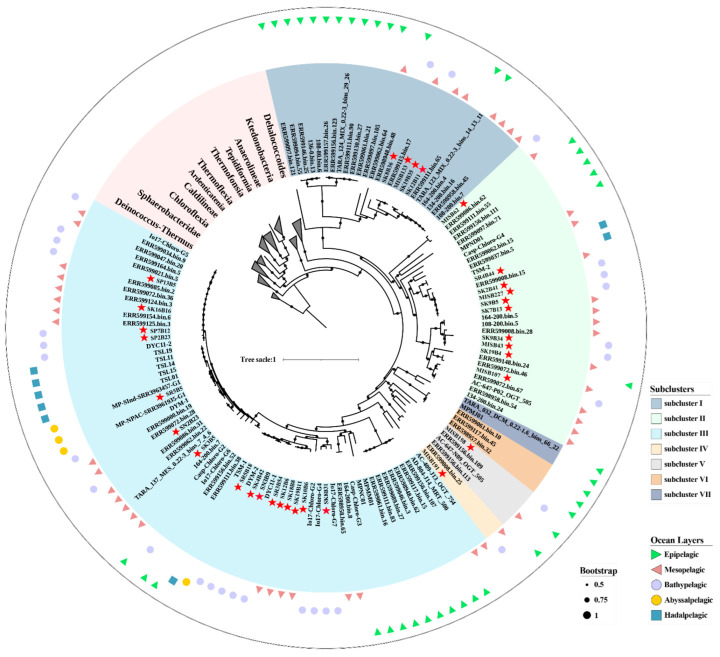
Maximum likelihood phylogenomic tree of SAR202. The phylogenomic tree was constructed based on the concatenated alignment of 43 commonly conserved proteins using IQ-Tree with model selection followed by tree inference based on 1000 replicates. The black dots on the branches denoted bootstrap support and only those >50% were depicted. The SAR202 MAGs in this study were highlighted with a red star. The information of reference genomes was listed in Table S2.

A total of 216.2 Gbp raw data obtained from the 12 MISNAC metagenomes were co-assembled to produce 5.1 Gbp contigs. Seven MAGs classified to SAR202 were obtained in this study and combined with the 25 SAR202 genomes curated from our library for southwestern Pacific water samples. A total of 32 SAR202 MAGs (completeness > 50%, contamination < 10%) were used to detect the transcriptional, temporal variations in SAR202 subclusters in this study (Table 1). To determine the taxonomic status of 32 SAR202 MAGs, the phylogenomic trees constructed with the 43 and 120 concatenated conserved proteins showed that those MAGs were assigned to seven subclusters (I–VII) in this study (Figure 2 and Figure S4). Most of the MAGs were affiliated with subclusters II and III (31.25% and 50%, respectively), among which four SAR202 MAGs belonged to subcluster I, 10 MAGs to subcluster II, 16 MAGs to subcluster III, one MAG to subcluster IV and one MAG to subcluster V. A total of 619 genomes were used to assess their taxonomic ranks based on the RED values (Table S3). Using this metric, SAR202 subclusters I, II and III were the domain groups and are best described as order level (Figure S5), which is similar to the taxonomic level based on the ANI result (Figure S6).

### 3.4. Transcriptional Activities of SAR202 Subclusters

**Figure 3:**
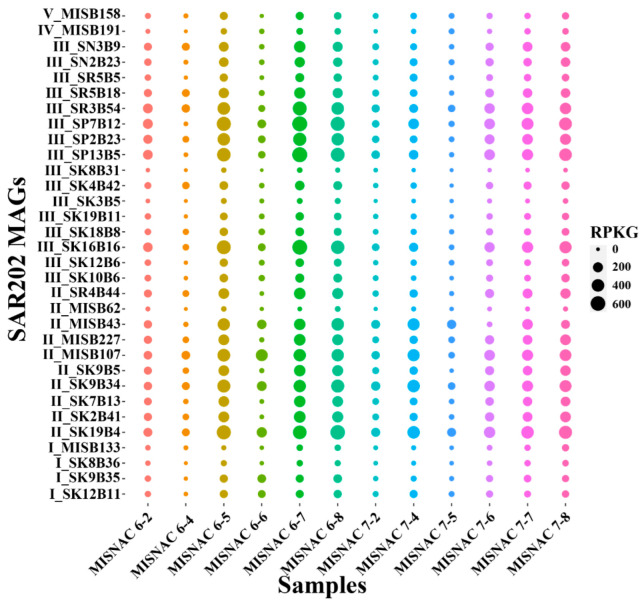
In situ transcriptional activities of SAR202 subclusters in the SCS. The dot size denotes the RPKG value, which reflects the in situ transcriptional activities of SAR202 species represented by different MAGs. The MAG IDs are initiated with a subcluster mark. The in situ samples were collected by MISNAC apparatus and the sampling time points are described in Table S1.

**Figure 4:**
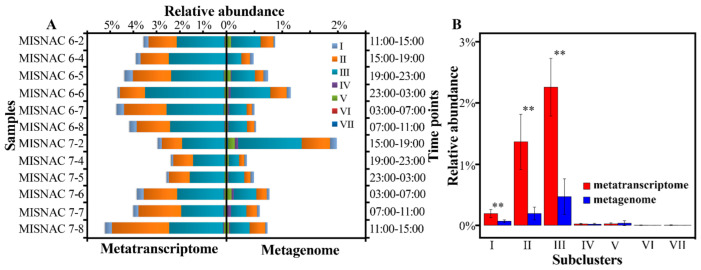
Relative abundance of SAR202 subclusters in metagenomic and metatranscriptomic data for different time periods. Twelve in situ microbial samples were continuously collected at 1022 m depth in the SCS by the MISNAC apparatus. (**A**) Percentages of 16S miTags of each SAR202 subcluster in all those extracted from metagenomes and metatranscriptomes are exhibited as their relative abundance. (**B**) The significant difference between metagenomics and metatranscriptomics data in relative abundance for each SAR202 subcluster was verified by *t*-test. ** represented a significant difference (*p* < 0.01).

In this study, 12 in situ metatranscriptomic raw data of 100.9 Gbp were obtained for our time-course samples (Table S1). The RPKG and TPM values of each SAR202 MAG were calculated as proxies to reflect their in situ transcriptional activities in the deep ocean (Figure 3 and Figure S7). SAR202 subclusters II and III were dominant in the metatranscriptomes of the SCS deep-sea environment (Figure 4A). Their transcriptional level peaked at 23:00–3:00 of day 1 and 11:00–15:00 of day 2. Seven genomes of SAR202 subcluster III (SN3B9, SN2B23, SR3B54, SP7B12, SP2B23, SP13B5, SK16B16) and eight genomes of SAR202 subcluster II (SR4B44, MISB43, MISB107, MISB227, SP9B5, SP7B13, SP2B41, SP19B4) could be mapped by a high percentage of transcriptomic reads (3.5% ± 1.1%). Among the MAGs, three SAR202 genomes (MISB43, MISB107, MISB227) were obtained by this study. Subclusters I, II and III were more abundant in metatranscriptomes, compared with their abundance in metagenomes with significant difference (*U*-test, *p* < 0.01), except for one time period (Figure 4B).

Comparing the SAR202 subclusters in the metagenomic and metatranscriptomic data, their relative abundance varied among sampling time intervals (Figure 4A), and subclusters I–III were more abundant in metatranscriptomes than in metagenomes with significant difference (*U*-test, *p* < 0.01) (Figure 4B). In both metagenomic data and metatranscriptomic data, SAR202 subclusters II and III were dominant in the deep-sea environment of the SCS, which is in agreement with our previous studies [11].

### 3.5. Functional Gene Transcripts in SAR202 Subclusters

**Figure 5:**
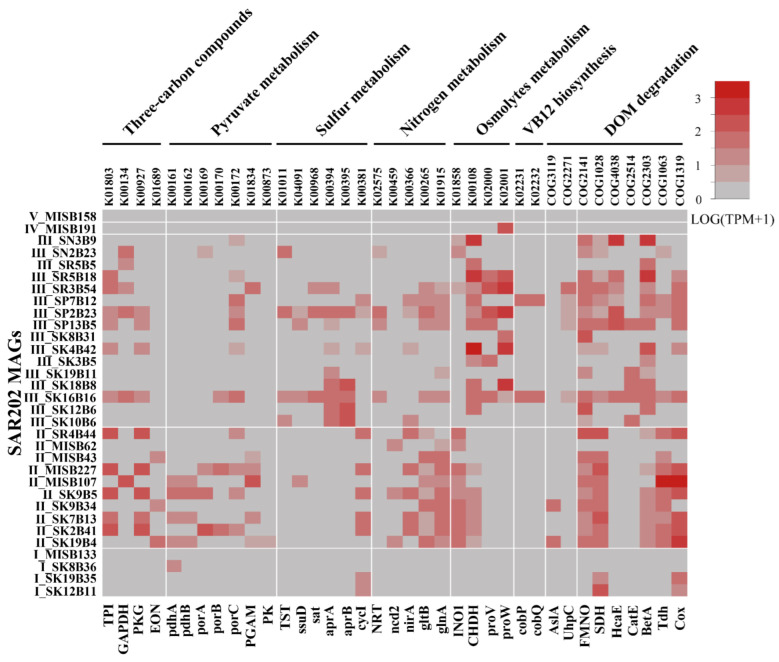
Transcriptional level of functional genes of SAR202 MAGs. For a functional gene, TPM values higher than 1 in three or more transcriptomic data from different time periods were averaged to estimate an overall transcriptional activity in a SAR202 species represented by the MAG. The gene names are shown in Table S5.

To further evaluate the activities of each SAR202 subcluster in the deep ocean, we explored the transcriptional levels of genes involved in basic metabolism (Figure 5). The expressional patterns of functional genes showed that the transcripts of genes involved in the metabolism pathway of three-carbon compounds during glycolysis, pyruvate metabolism and the TCA cycle were detected in SAR202 subclusters II and III. The transcripts of genes encoding triosephosphate isomerase (TPI), glyceraldehyde 3-phosphate dehydrogenase (GAPDH), phosphoglycerate kinase (PGK), pyruvate ferredoxin oxidoreductase (*porA*, *porB* and *porC*), and glycerate phosphomutase (PGAM) were detected in SAR202 subclusters II and III. However, transcripts of genes encoding pyruvate dehydrogenase (*pdhA* and *pdhB*) and pyruvate kinase (PK) were only detected in SAR202 subcluster II, and transcripts of genes encoding enolase (ENO) were only detected in subcluster III (Tables S4 and S5).

The transcripts of genes encoding enzymes involved in DOM degradation pathways, such as F420-dependent N5, N10-methylene tetrahydromethanopterin reductase and related flavin-dependent oxidoreductases (FMNO), short-chain alcohol dehydrogenases (SDH) and aerobic-type carbon monoxide dehydrogenase (Cox) were detected in SAR202 subclusters II and III. Among them, the expression level of the genes encoding short-chain alcohol dehydrogenases in SAR202 subcluster II was higher than that in other subclusters, while the transcripts of genes encoding phenylpropionate dioxygenase and related ring-hydroxylating dioxygenases (HcaE) and ring-cleavage extradiol dioxygenase (CatE) were only detected in SAR202 subcluster III. In addition, the transcripts of genes encoding arylsulfatase A or related enzyme protein (AslA) were only found in SAR202 subcluster II, and the transcripts of genes encoding sugar phosphate permease (UhpC) were only encoded by the MAGs from SAR202 subcluster III (Figure 5, Tables S4 and S5).

In the deep-sea sulfur metabolism pathways, the transcripts of genes encoding sulfite reductase (*cysI*), alkanesulfonate monooxygenase (*ssud*) and rhodanese (TST) were assigned to SAR202 subclusters II and III, while those encoding enzymes involved in the reverse sulfate reduction pathway such as sulfate adenylyltransferase (*sat*) and transcripts of adenylyl-sulfate reductase A/B (*aprA/B*) were only detected in SAR202 subcluster III (Figure 5, Tables S4 and S5). Associated with the deep-sea nitrogen metabolism pathways, genes encoding nitrite reductase (*nirA*) and glutamine synthetase (*glnA* and *gltB*) were transcribed by SAR202 subclusters II and III. The nitronate monooxygenase gene (*ncd2*) was only transcribed in SAR202 subcluster II, while the transcripts of genes encoding MFS nitrate/nitrite transporter (NNP family, nitrate/nitrite transporter NRT) were only detected in SAR202 subcluster III (Figure 5, Tables S4 and S5).

For the utilization of osmotic pressure-regulating substances in the deep-sea environment, the transcripts of genes encoding *myo*-inositol-1-phosphate synthase (INO1) and choline dehydrogenase (CHDH) were detected in SAR202 subclusters II and III. The expression level of INO1 in SAR202 subcluster II was higher than that in SAR202 subcluster III, while the transcripts of the genes encoding glycine betaine/proline transporter (ProV and ProW) were only present in SAR202 subcluster III transcriptomes (Figure 5, Tables S4 and S5). The genes involved in the vitamin B12 (VB12) synthesis pathway, including those encoding adenosylcobinamide kinase (CobP) and adenosylcobyric acid synthase (CobQ) were only found in metagenomics and metatranscriptomics data of SAR202 subcluster III (Figure 5, Tables S4 and S5). Interestingly, the expression levels of the functional genes were variable with sampling time periods (Figure S8).

## 4. Discussion

### 4.1. Phylogenetic Analysis of SAR202

Seven subclusters in SAR202 have been discerned previously [17,18,19]. In this study, we reported phylogenomics relationships of SAR202 with more genomes and found that subclusters I, II and III might be classified as orders of SAR202 based on the RED and ANI values. The taxonomic level of the other four subclusters could not be evaluated, probably due to the small number of genomes available for RED calculations. The phylogenetic analysis indicated that a number of genomes should be accounted for RED re-analysis, and taxonomic changes would be required to accommodate the reclassification of SAR202 subclusters.

### 4.2. Time-Course Changes in SAR202 Subclusters

The contribution of SAR202 to material cycles in the deep ocean has been underestimated by metagenomic data alone [11]. In addition, we first found that SAR202 subclusters also differed in their relative abundance and had fluctuations in transcriptional activities and functional gene expression patterns, which were likely related to the varying deposition rates of organic matter from sinking particulate carbon in the sampling site, and was not reported in the previous studies [17,18,19].

### 4.3. Carbon Sources for Each SAR202 Subcluster

In previous studies, the characteristics of SAR202 MAGs and their roles in the circulation of deep-sea material were discussed [16,18,20]. However, due to the influence of sampling technology and the limited number of SAR202 genomes, the differences in the deep-sea ecological functions of each SAR202 subcluster have not been further assessed. In this study, the in situ DNA and RNA samples obtained by the MISNAC apparatus in the SCS deep-sea environment were first used to address this issue. Based on in situ metagenomic and metatranscriptomic analysis, we performed a deep comparison of the contributions of five SAR202 subclusters represented by 32 SAR202 MAGs in the biogeochemistry and nutrient cycles in the deep-sea environment. The highest transcriptional activities of SAR202 subclusters II and III at a depth of ~1000 m in the SCS showed that they were the most active and dominant groups in the deep-sea environment as shown in previous studies [5,11].

Given the high abundance of SAR202 subclusters II and III in the global deep-sea water, they are most important among the SAR202 subclusters in the deep ocean [15]. Although a complete three-carbon metabolic pathway was present in the genomes of SAR202 subclusters I–V, only transcripts of those genes in SAR202 subclusters II and III were detected in the metatranscriptomic data. The three-carbon pathway was the component of glycolysis degrading the glucose to pyruvate and generating ATP, which might further hint that SAR202 subclusters II and III are the main active groups in the deep-sea environment. Arylsulfatase A or related enzyme could be used to degrade sulfated polysaccharides from marine surface algae [18]. Their transcripts implied that SAR202 subcluster II might utilize sulfated polysaccharides from upper water as a carbon source in the deep-sea environment, while the expression of sugar phosphate permease protein could help SAR202 subcluster III absorb phosphopolysaccharides from the deep-sea environment as a carbon source [43]. We speculated that the types and sources of polysaccharides utilized by SAR202 subclusters II and III were different in the deep-sea environment.

F420-dependent N5, N10-methylene tetrahydromethanopterin reductase and related flavin-dependent oxidoreductases proteins could catalyze the insertion of oxygen into the semi-unstable alicyclic ring, and then participate in the degradation of DOM in the deep-sea environment [15,17]. The high expression level of those genes in SAR202 subclusters II and III indicated that the two SAR202 subclusters had important contributions to DOM degradation in the deep-sea environment and the efficiency of the microbial ‘carbon pump’ [44]. The short-chain alcohol dehydrogenase protein could catalyze the oxidation of short-chain fatty alcohol [15,17]. Thus, SAR202 subcluster II, with a higher transcript level of the gene, would play important roles in the utilization of short-chain alkanes in agreement with previous studies [17]. Phenylpropionate dioxygenase and related ring-hydroxylating dioxygenases could be used to metabolize monocyclic or polycyclic aromatic compounds released by phytoplankton in the surface ocean and provide energy and carbon sources for SAR202 cells [16,18,19]. The ring-cleavage extradiol dioxygenase protein could be further annotated as catechol 2,3-dioxygenase, which was one of the most important enzymes in the degradation pathway of aromatic compounds, and could catalyze catechol to 2-hydroxymuconate-6-semialdehyde [45,46]. The transcripts of those genes were only detected in SAR202 subcluster III, suggesting that subcluster III was one of the important consumers for refractory organic carbon in the deep sea as reported in previous studies [17,18]. Therefore, we propose that different carbon sources preferentially selected by SAR202 subclusters II and III might be a strategy for survival in the deep sea, which was not reported by the previous studies [15,17,18,19].

### 4.4. Contribution of SAR202 Subclusters in Sulfur Metabolism

In this study, the transcripts of genes encoding rhodanese were only detected in SAR202 subcluster III, which suggests that SAR202 subcluster III might be the main user of thiosulfate in the deep-water environment and degrade it to sulfite as demonstrated previously [47,48]. The genes encoding alkanesulfonate monooxygenase were only found in the SAR202 subclusters II and III genomes with high transcriptional levels, indicating that the two subclusters could further degrade methanesulfonate to formaldehyde and sulfite [15]. The genes involved in the reverse dissimilatory sulfite pathway were only identified and expressed in SAR202 subcluster III, and the genes catalyzing the further reduction in sulfite to hydrogen sulfide were absent in the SAR202 subcluster III genomes. These results imply that SAR202 subcluster III might obtain sulfite produced by the degradation of deep-sea sulfide (thiosulfate and methanesulfonate) and oxide sulfite to produce sulfate and ATP through the reverse dissimilatory sulfite pathway in agreement with previous studies [15,19]. Conversely, although no complete assimilation sulfate reduction and dissimilatory sulfite pathways were found in the SAR202 subcluster II genomes, the genes encoding sulfite reductase were identified and expressed, indicating that SAR202 subcluster II might use sulfite produced by the degradation of methanesulfonate for the synthesis of cysteine [15]. In addition, a complete pathway of assimilation sulfate reduction was identified in the SAR202 subcluster I genomes, and no transcripts of those genes were detected, suggesting that SAR202 subcluster I might not be the main consumer of sulfate in the deep-sea environment. Considering that the transcripts of genes involved in DOM degradation were lower in the subcluster III than those in subcluster II, this indicates that ATP produced by the reverse dissimilatory sulfite pathway is a vital productivity mode for SAR202 subcluster III cells in the deep ocean as reported in previous studies [19]. We can infer that the contributions of SAR202 subclusters are different in the sulfur metabolism in the deep-sea, and the deep-sea sulfite might be more important for SAR202 subcluster III cells.

### 4.5. Roles of Each SAR202 Subcluster in Nitrogen Metabolism

Interestingly, only the transcripts of genes encoding MFS nitrate/nitrite transporter were detected in SAR202 subcluster III, indicating that SAR202 subcluster III might directly absorb nitrite from the environment in the deep sea [15,49]. The gene encoding nitrite monooxygenase could oxide the nitroalkane to nitrite, and was identified in SAR202 subclusters II and III genomes. However, the gene only transcribed in the SAR202 subcluster II, hinting that the deep-sea nitroalkanes might be important nitrogen sources for SAR202 subcluster II. In addition, the transcripts of genes encoding nitrite reductase were detected in SAR202 subclusters II and III, which could catalyze the reduction of nitrite to ammonia and enter the amino acid metabolism under the action of glutamine synthetase [15,49]. It was found that the roles in the nitrite metabolism of different SAR202 subclusters were also different in the deep ocean, and the low content of nitrite in the water unveiled that SAR202 subcluster II might have more important roles in the deep-sea nitrogen cycle.

### 4.6. Choice of Osmolytes by Each SAR202 Subcluster

Osmolytes such as *myo*-inositol, glycine and betaine were critical for the survival of deep-sea organisms as they confront extreme hydrostatic pressure [16,50,51]. In this study, the adaptability mechanisms of each SAR202 subcluster in the deep-sea environment were explored for the first time by using metatranscriptomic data. To adapt to the extremely high hydrostatic pressures in the deep sea, *myo*-inositol as an osmolyte could be produced by glucose degradation and accumulated in cells [15,52]. The genes involved in inositol biosynthesis were all present in the SAR202 subclusters I–V genomes, but the key *myo*-inositol synthesis genes encoding *myo*-inositol-1-phosphate synthase were only expressed in SAR202 subclusters II and III. Moreover, the accumulation of betaine in cells might also be important for tolerance against hydrostatic pressure [15]. The expression results of functional genes showed that SAR202 subclusters II and III could not only be obtained from the environment through transport proteins but also produced by the degradation of choline in the deep-sea environment, which suggests that betaine might be an important source of osmolytes in SAR202 subclusters II and III cells in the deep-sea environment [16,18,20]. Interestingly, the expression levels of genes encoding *myo*-inositol-1-phosphate synthase in SAR202 subcluster II were higher than those in subcluster III, while the genes involved in cholinergic degradation were the opposite, which indicates that the two SAR202 subclusters might prefer different osmolytes to resist high hydrostatic pressures in the deep-sea environment.

### 4.7. Metabolism of Other Substances

Although we obtained more SAR202 genomes classified into seven subclusters (I–VII) in this study, the VB12 synthesis pathway was only found in the SAR202 subcluster III genomes. The transcriptional data also confirmed that the genes involved in the pathway were active in SAR202 subcluster III. It was speculated that SAR202 subcluster III might be an important producer of VB12 in the deep-sea environment [15]. In addition, different from our previous studies, due to the number of SAR202 genomes increasing, the genes encoding chitinase were identified in genomes of both SAR202 subclusters II and III, which implied that chitin might be an important source of carbon and nitrogen in the deep-sea environment not only for SAR202 subcluster II but also for SAR202 subcluster III [15]. However, the transcripts of genes encoding chitinase were not detected in the transcriptional data.

## 5. Conclusions

In this study, the application of the MISNAC device to obtain in situ nucleic acid samples helped us to further explore the compositions and re-evaluate the ecological functions of dominant microorganisms in the deep-sea environment. The analysis of the response mechanisms of functional genes using in situ omics data proved that SAR202 subclusters II and III are the active groups and play important roles in deep-sea material cycles. The investigations into the ecology, genomics and transcriptomics of the deep-sea in situ metagenomics and metatranscriptomics data have cast a light on the mechanisms that foster the diversification of the SAR202 subclusters. In addition, because the MISNAC apparatus was limited by the sampling environment, we have not yet obtained definite conclusions on the temporal scale of the periodic changes in the structure of deep-sea microbial communities and the functional gene expression patterns of the deep-sea dominant groups. Future studies on the circadian rhythms of deep-sea microorganisms are needed with more support with regards to environmental factors and in situ cultivations.

## Figures and Tables

**Table 1:** Genomic information of SAR202 MAGs in this study.

Genome ID	Depth (m)	Subcluster	Longitude (°E)	Latitude (°N)	Genome Size (bp)	GC (%)	CDS Number	Coding Density	Compl (%)	Conta (%)
MISB158	1022	V	110.01	17.06	1,133,699	0.46	1299	0.88	50.69	6.89
MISB191	1022	IV	110.01	17.06	2,050,173	0.56	2106	0.87	95.38	2.97
SN3B9	1022	III	114.99	11.97	2,219,140	0.58	2398	0.85	53.21	3.47
SN2B23	2000	III	116.49	11.99	2,277,881	0.59	2254	0.87	78.88	1.98
SR5B5	4210	III	123.03	22.19	3,297,532	0.59	3180	0.86	95.71	0.99
SR5B18	4210	III	123.03	22.19	3,974,242	0.58	3976	0.85	93.62	3.96
SR3B54	1646	III	120.04	21.87	2,388,874	0.58	2416	0.85	77.43	2.15
SP7B12	1000	III	110.17	17.19	1,461,202	0.57	1616	0.85	80.77	0.99
SP2B23	1000	III	110.18	17.19	2,554,614	0.58	3004	0.87	57.49	4.13
SP13B5	1000	III	110.17	17.19	1,973,705	0.57	2212	0.87	55.05	7.13
SK8B31	3000	III	118.17	13.96	2,810,028	0.56	2809	0.84	67.21	0.61
SK4B42	3000	III	116.48	18.01	2,736,876	0.58	2931	0.86	52.55	1.75
SK3B5	200	III	116.48	18.01	2,415,447	0.55	2531	0.87	82.84	4.76
SK19B11	500	III	118.2	16	3,020,336	0.57	3218	0.85	89.19	3.3
SK18B8	500	III	118.18	14.98	2,560,045	0.57	2812	0.86	91.75	2.48
SK16B16	500	III	114.99	9.99	2,946,073	0.57	2930	0.86	79.07	2.67
SK12B6	500	III	114.99	11.97	2,493,422	0.57	2749	0.87	81.66	1.52
SK10B6	600	III	116.49	14	1,833,071	0.57	1968	0.86	56.46	9.03
SR4B44	6000	II	123.31	22.82	2,606,463	0.56	3020	0.86	98.68	0
MISB62	1022	II	110.01	17.06	1,047,704	0.3	1167	0.94	93.73	2.97
MISB43	1022	II	110.01	17.06	1,446,211	0.42	1609	0.92	67.87	1.65
MISB227	1022	II	110.01	17.06	3,068,488	0.56	3200	0.86	61.73	1.1
MISB107	1022	II	110.01	17.06	1,845,114	0.46	1919	0.9	78.11	6.53
SK9B5	1000	II	116.49	14	2,676,491	0.56	2880	0.86	96.7	1.1
SK9B34	1000	II	116.49	14	1,950,012	0.43	2185	0.9	92.24	1.98
SK7B13	850	II	118.19	16.66	2,301,923	0.56	2574	0.86	61.95	0
SK2B41	2000	II	116.48	18.01	3,383,583	0.56	3501	0.85	82.84	4.76
SK19B4	500	II	118.2	16	2,083,146	0.43	2022	0.92	68.96	5.94
MISB133	1022	I	110.01	17.06	2,386,853	0.58	2217	0.85	92.65	2.4
SK8B36	3000	I	118.17	13.96	2,329,958	0.58	2238	0.85	69.08	0
SK19B35	500	I	118.2	16	1,219,668	0.47	1243	0.9	68.28	1.98
SK12B11	500	I	114.99	11.97	1,660,536	0.47	1736	0.9	54.32	2.6

## Data Availability

The 32 assembled SAR202 MAGs have been deposited to CNGB and can be accessed under the BioProject CNP0002642. The raw sequencing data from the current study are not publicly available but are available from the corresponding author on reasonable request.

## Supplementary Materials

The following supporting information can be downloaded at: https://www.mdpi.com/article/10.3390/microorganisms10081629/s1.
