# Supplementary material for: In Situ Genomics and Transcriptomics of SAR202 Subclusters Revealed Subtle Distinct Activities in Deep-Sea Water
Source: Microorganisms. 2022 Aug 12;10(8):1629. doi: 10.3390/microorganisms10081629 (PMC9416657; doi:10.3390/microorganisms10081629)
Supplement: Supplementary file 1 [file microorganisms-10-01629-s001.zip › microorganisms-1809329-supplementary figures.pdf]

In situ genomics and transcriptomics of SAR202 subclusters revealed subtle distinct activities in deep-sea water

Zhan-Fei Wei[2], Qing-Mei, Li[3], Rui Lu[3], Peng-Fei Zheng[3], Yong Wang[1, 3*]

[1] *Shenzhen International Graduate School, Tsinghua University, Shenzhen, P.R. China*

[2] *Yellow Sea Fisheries Research Institute, Chinese Academy of Fishery Sciences, Qingdao, Shandong, P.R. China*

[3] *Institute of Deep-Sea Science and Engineering, Chinese Academy of Sciences, Sanya, Hainan, P.R. China*

[*]**Corresponding author:**

Yong Wang, PhD

Shenzhen International Graduate School, Tsinghua University

No. 19, Gaoxinnanqi Road, Shenzhen, Guang Dong, P.R. of China

Phone: 086-898-88381062

E-mail: wangyong@sz.tsinghua.edu.cn

Running title: *In situ* activities of SAR202 subclusters

Keywords: microbial community, SAR202, DOM, omics data, MISNAC

Figure S1: Profile of depth, temperature, dissolved oxygen, and salinity during lander deployments in the SCS.

A: The first dive; B: The second dive. The black star represents the time point at which the MISNAC apparatus was initiated at 1,022 m depth in the SCS.

Figure S2: Microbial community structures at phylum level.

The 16S rRNA gene fragments (16S miTags) with length >100 bp were extracted from the metagenomic and metatranscriptomic data, respectively. Classification of 16S miTags was used to illustrate microbial communities of metagenomes (A) and metatranscriptomes (B) at phylum level. The DNA/RNA samples were collected at 1,022 m depth in the SCS by MISNAC apparatus. The sampling time periods were described in Table S1.

Figure S3: Microbial community structures at order level.

The 16S rRNA gene fragments (16S miTags) with length > 100 bp were extracted from the metagenomic and metatranscriptomic data, respectively. Classification of 16S miTags was used to illustrate microbial communities of metagenomes (A) and metatranscriptomes (B) at order level. The DNA/RNA samples were collected at 1,022 m depth in the SCS by MISNAC apparatus. The samples were described in Table S1.

Figure S4: Phylogenomic position of SAR202 among Bacteria.

The phylogenomic tree was based on 120 concatenated bacteria marker gene using FastTree with the GTR + GAMMA model. The SAR202 MAGs in this study were highlighted with a black star. The information of bacteria genomes was listed in Table S3.

Figure S5: Information of relative evolutionary divergence (RED) of SAR202 subcluters.

The relative evolutionary divergence represented all internal tree nodes with assigned taxonomic ranks. Rank assignments were based on the Genome Taxonomy Database (GTDB).

Figure S6: ANI plotting of SAR202 genomes.

A total of 130 SAR202 MAGs in this study were used to calculate the pairwise ANI value. The information of the SAR202 genomes was listed in Table S2.

Figure S7: TPM value of each SAR202 MAG at sampling time.

Summation of gene TPM values in the same bin. The *in situ* samples were collected by MISNAC apparatus and the sampling time points were described in Table S1.

Figure S8: Fluctuation of functional gene expression

Summation of CO TPM values. The expression levels of functional genes were listed in Table S4.

Table S1: Metagenomes and metatranscriptomes from MISNAC samples collected at the ~1,000 m depth of the SCS.

Table S2: List of genomes used for the phylogenomic tree of SAR202.

Table S3: Information of bacteria genomes used for calculating RED value and phylogenomic tree.

Table S4: Transcriptional levels of functional genes in SAR202 subclusters in this study.

TPM value greater than 1 in three or more transcriptome data were averaged to estimate transcriptional level of functional genes.

Table S5: Information of the functional genes of SAR202.
