# Supplementary figures and images for: In Situ Genomics and Transcriptomics of SAR202 Subclusters Revealed Subtle Distinct Activities in Deep-Sea Water
Source: Microorganisms. 2022 Aug 12;10(8):1629. doi: 10.3390/microorganisms10081629 (PMC9416657; doi:10.3390/microorganisms10081629)

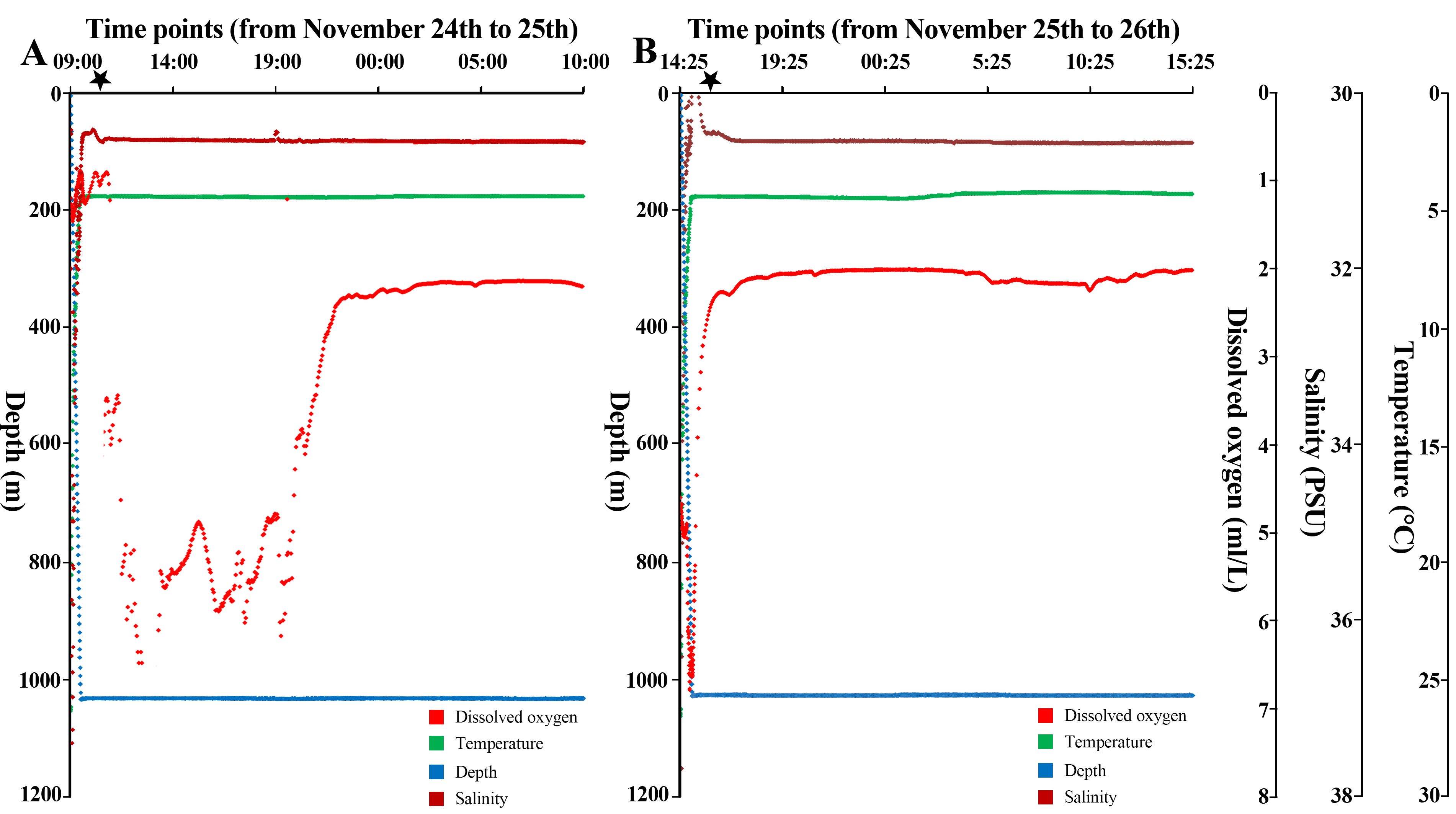

Supplement: Supplementary file 1 [file microorganisms-10-01629-s001.zip › Figure S1.tif]

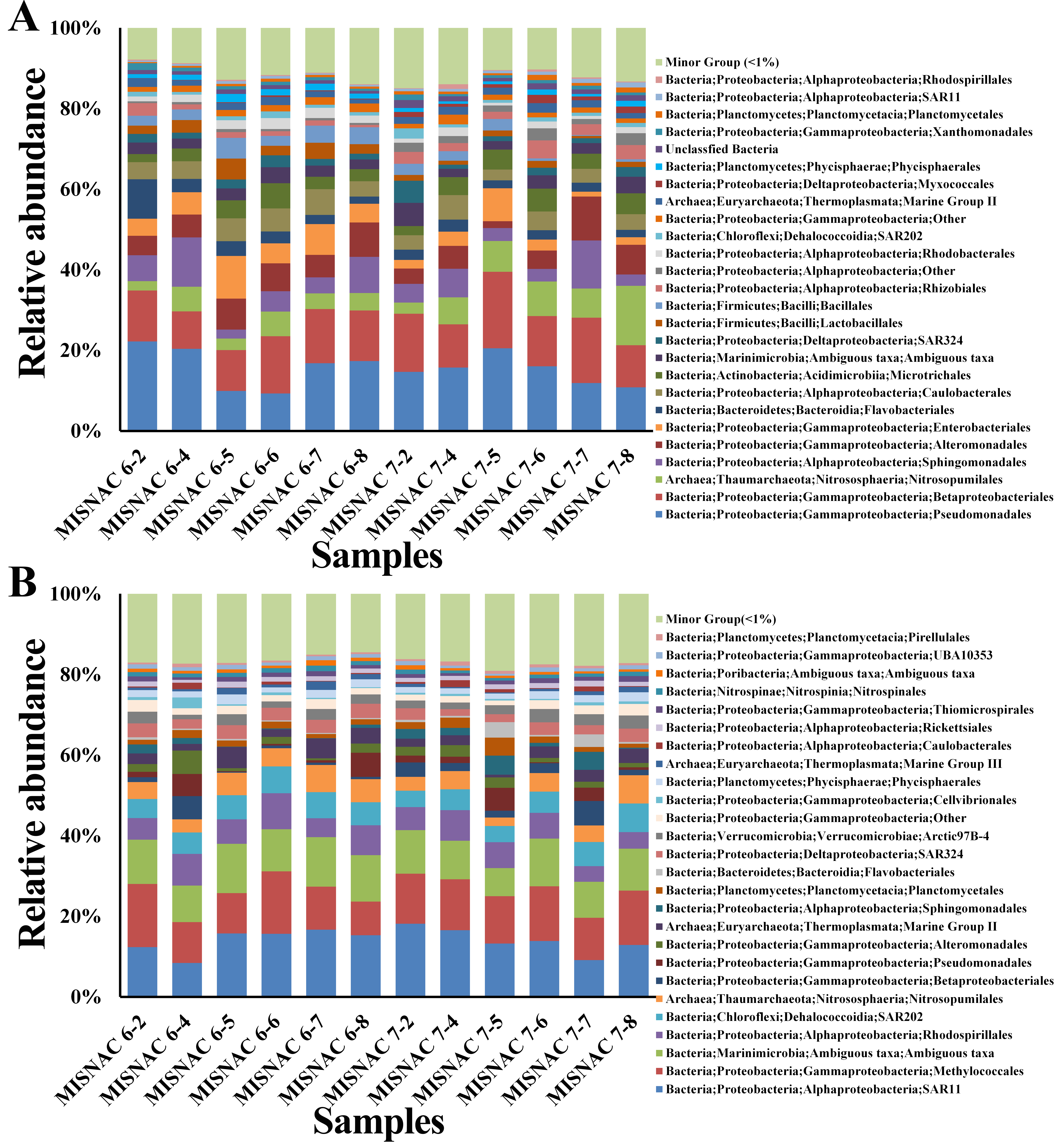

Supplement: Supplementary file 1 [file microorganisms-10-01629-s001.zip › Figure S2.tif]

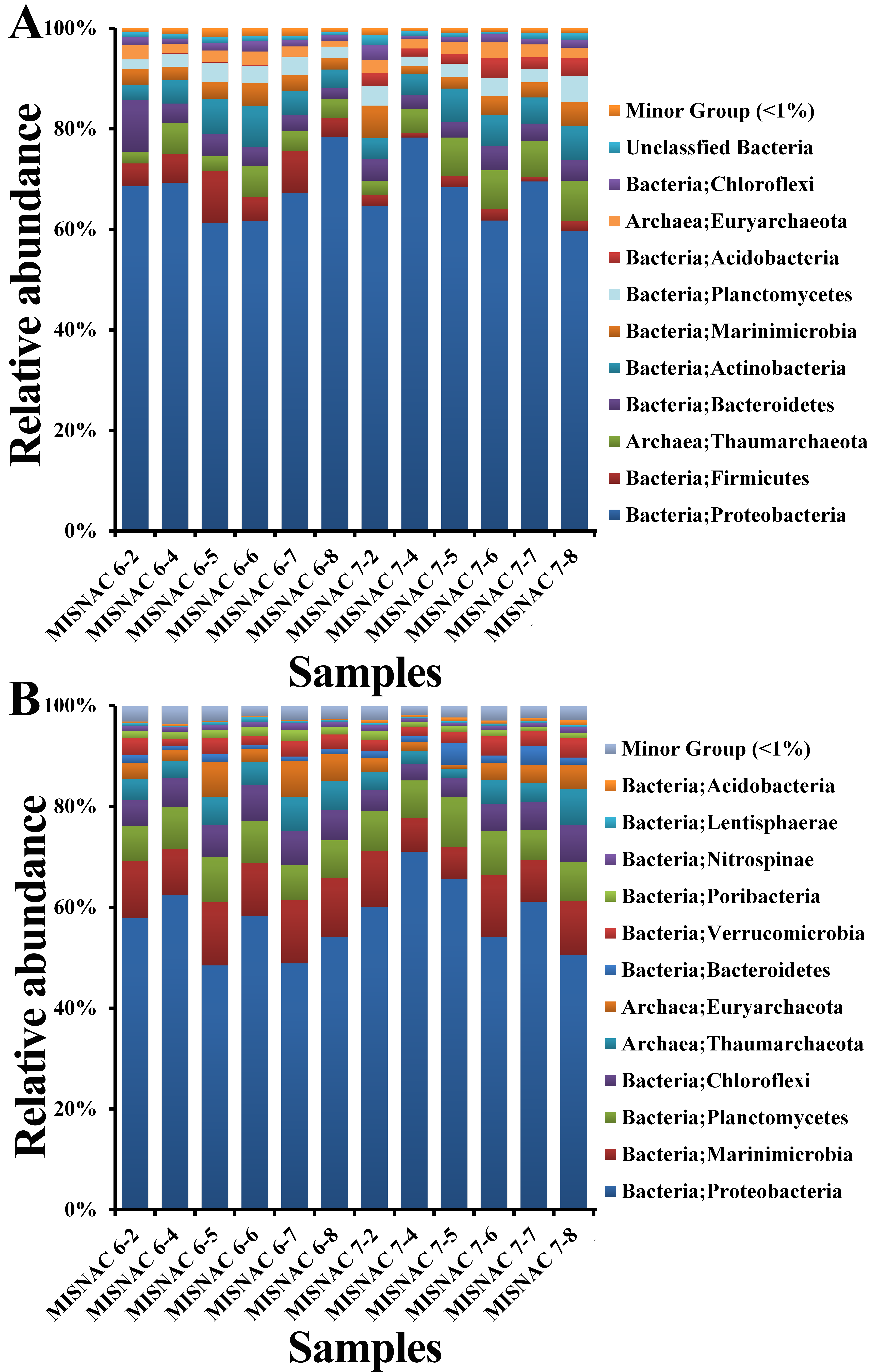

Supplement: Supplementary file 1 [file microorganisms-10-01629-s001.zip › Figure S3.tif]

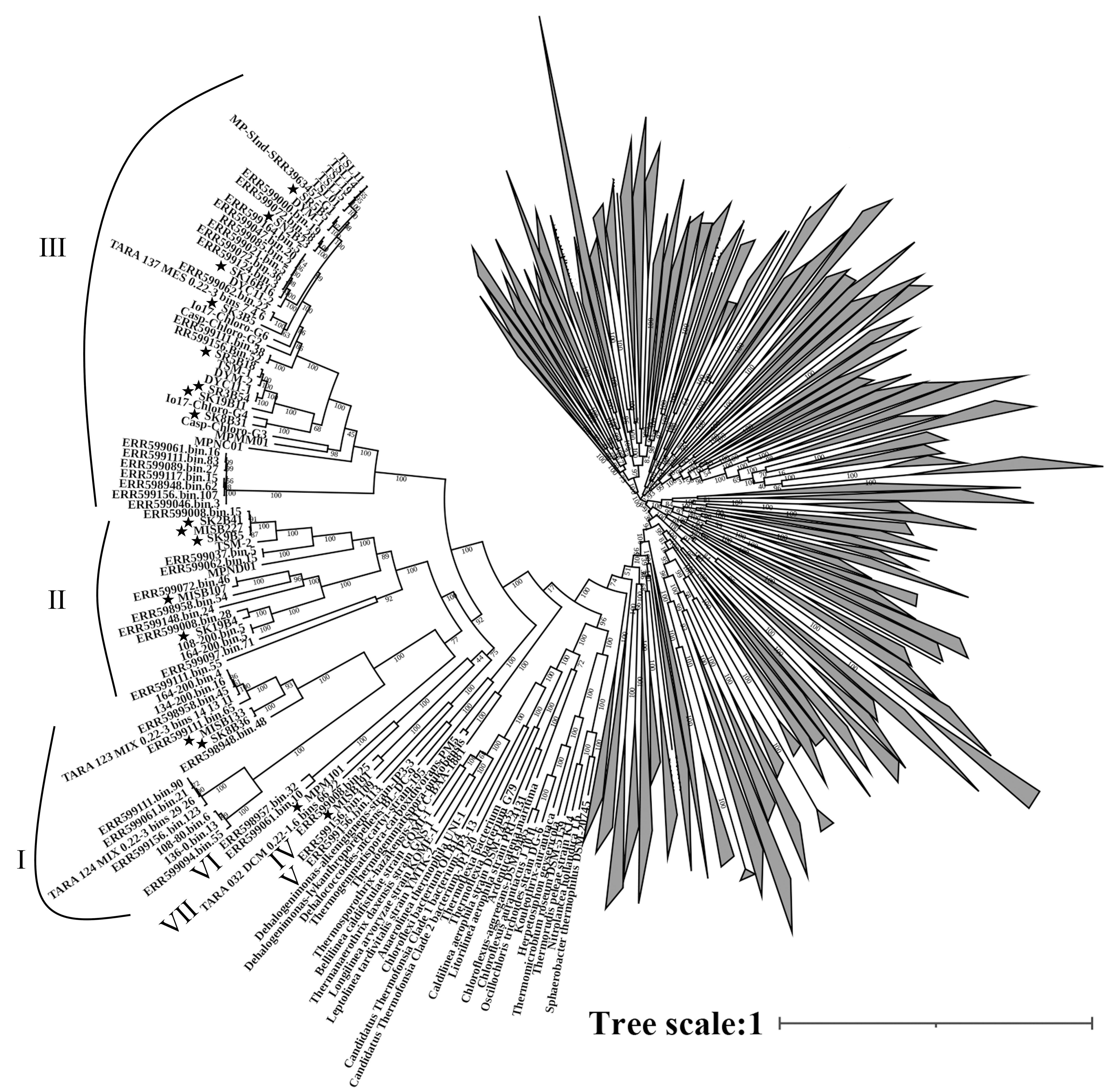

Supplement: Supplementary file 1 [file microorganisms-10-01629-s001.zip › Figure S4.tif]

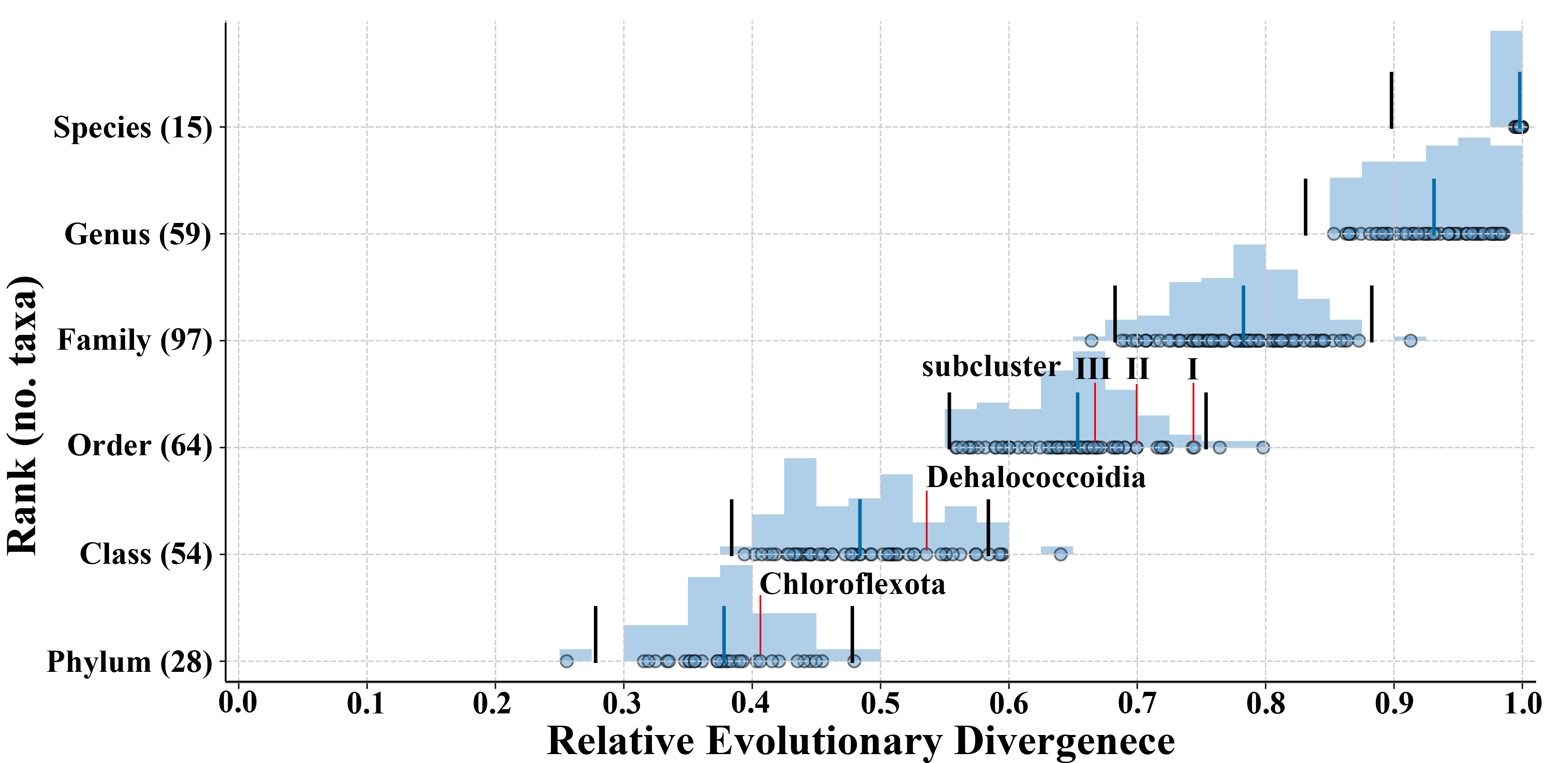

Supplement: Supplementary file 1 [file microorganisms-10-01629-s001.zip › Figure S5.tif]

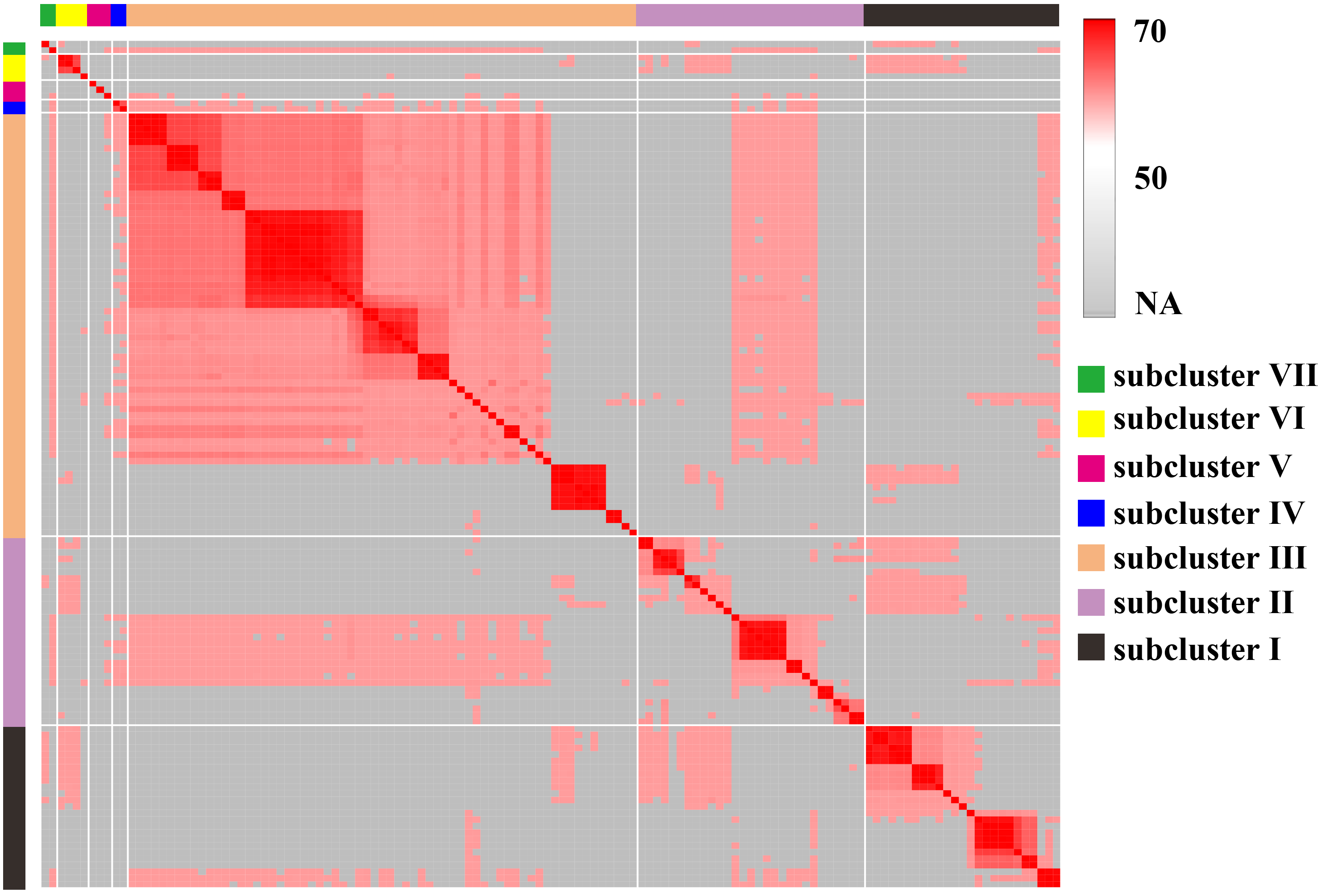

Supplement: Supplementary file 1 [file microorganisms-10-01629-s001.zip › Figure S6.tif]

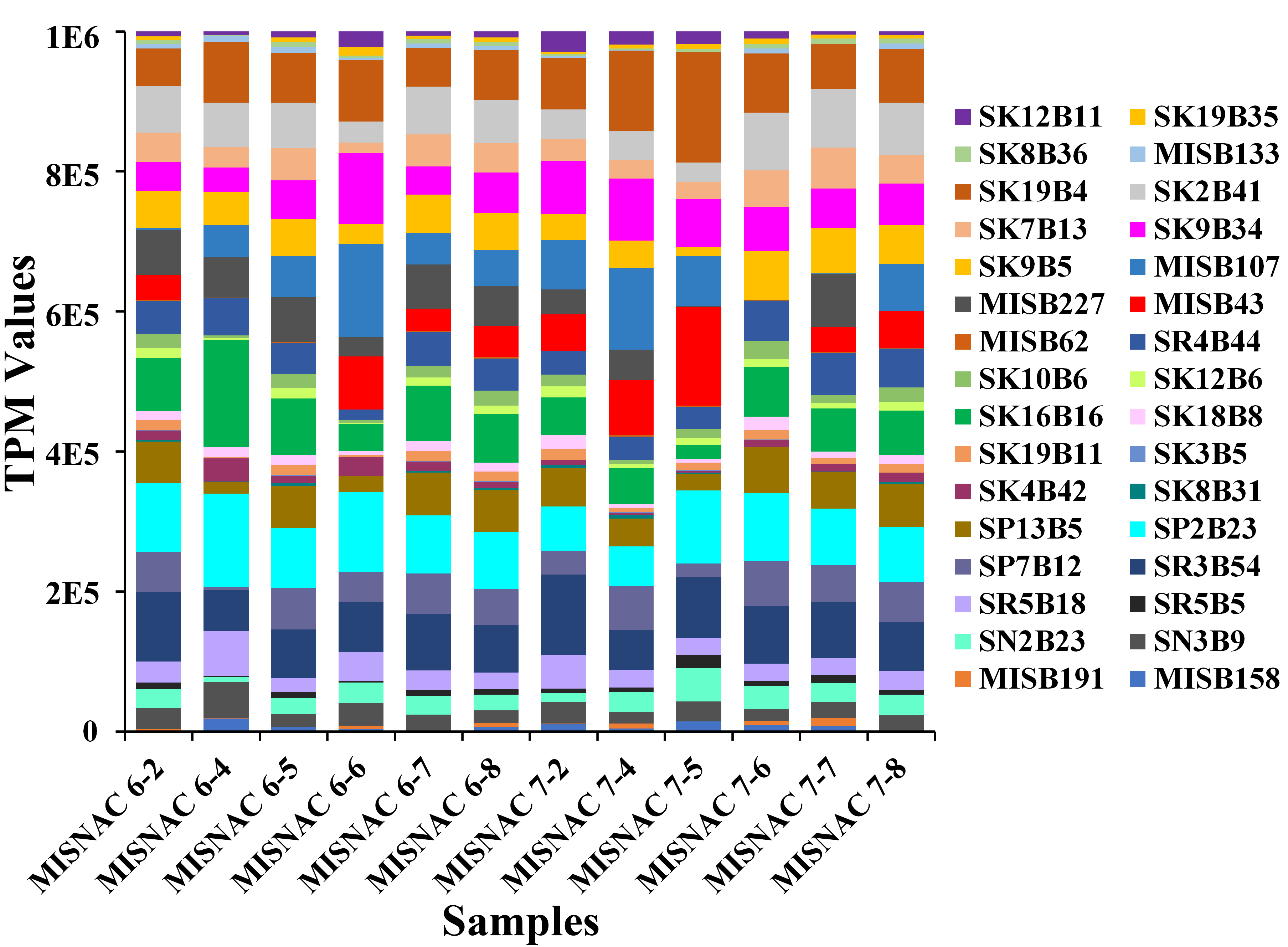

Supplement: Supplementary file 1 [file microorganisms-10-01629-s001.zip › Figure S7.tif]

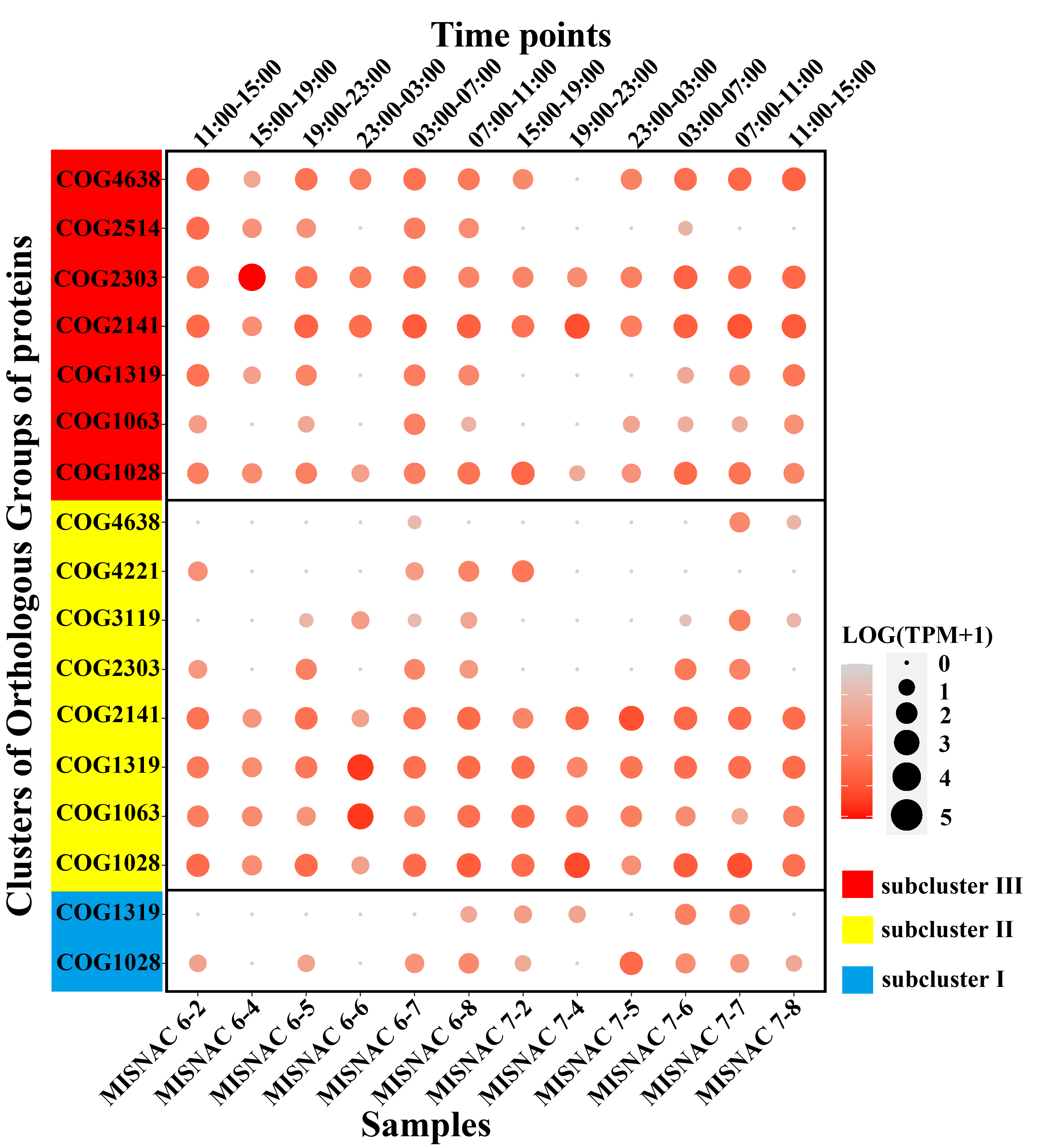

Supplement: Supplementary file 1 [file microorganisms-10-01629-s001.zip › Figure S8.tif]
